# Invitation for FOBt screening and colorectal cancer mortality: A prospective analysis in the Million Women Study cohort

**DOI:** 10.1002/ijc.35437

**Published:** 2025-04-11

**Authors:** Roger G. Blanks, Rupert J. Alison, Gillian K. Reeves

**Affiliations:** ^1^ Cancer Epidemiology Unit University of Oxford Oxford UK

**Keywords:** colorectal cancer, mortality, screening

## Abstract

Using linked data from the Million Women Study (MWS) cohort and the NHS Bowel Cancer Screening Programme (NHS BCSP) offering biennial guaiac faecal occult blood test (gFOBt) screening from 2006, we examined factors associated with screening acceptance, and differences in colorectal cancer (CRC) mortality by screening invitation status. Characteristics of attenders and non‐attenders were compared among 752,007 MWS participants born 1940–1950, who were all invited for at least one round of routine screening. Women declining screening had higher deprivation and smoking levels, and a 2‐fold risk of all‐cause and CRC mortality compared with women who accepted. Of 246,160 women born in 1935–1939, 111,956 were assigned to the “no intention to invite” group, and 134,204 to the “intention to invite” group based on year of birth and postcode sector, with an average of 0.01 and 2.40 screening invitations in each group, respectively. During a mean follow up of 11.9 years, there were 858 and 791 CRC deaths in the “intention to invite” and “no intention to invite” groups, respectively. In the period 4 or more years after study entry there was no significant reduction in risk of death from CRC associated with invitation for screening (RR = 0.94, 95%CI 0.83–1.06), but evidence of differences in associations by anatomical sub‐site, with a reduction in deaths from distal colon cancer (0.64, 0.47–0.88), but not proximal (1.02, 0.83–1.25) or rectal cancer (0.97, 0.79–1.20) (*p*‐value for heterogeneity by subsite = 0.05). Investigation of the effectiveness of current bowel screening methods using faecal immunochemical testing (FIT) by sex and cancer sub‐site is warranted.

## Graphical abstract

What's new?

The screening effectiveness of population screening programmes can differ from the screening efficacy measured in randomised controlled trials and is often difficult to measure directly. The authors used data on a subset of women born between 1935 and 1939 from the Million Women Study to estimate the effect of screening on colorectal cancer mortality using a quasi‐randomised trial design. There was no clear reduction in the risk of death from colorectal cancer associated with invitation for screening. Differences in associations were observed by anatomical sub‐site, with a reduction in deaths from distal but not proximal colon or rectal cancer.

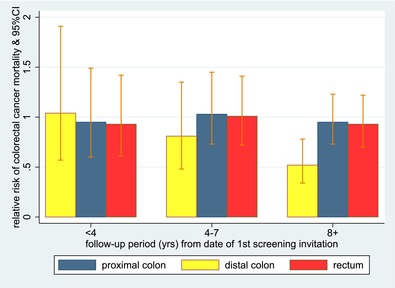

**Abbreviations**
BMI: body mass index
CRC: colorectal cancer
FIT: faecal immunochemical test
gFOBt: Guaiac faecal occult blood test
HRT: hormone replacement therapy
MWS: Million Women Study
NHS: National Health Service
NHS BCSP: National Health Service Bowel Cancer Screening Programme
RCT: randomised controlled trial

## 1 INTRODUCTION

The NHS Bowel Cancer Screening Programme (NHS BCSP) was rolled out gradually within the UK between 2006 and 2010. It originally offered 2 yearly (biennial) postal guaiac faecal occult blood test (gFOBt) screening for men and women registered with a GP aged 60–69 years and, from 2010, screening was gradually extended to ages 70–74 years. Individuals who tested positive based on the gFOBt screen were referred for a full colonoscopy. Those who did not participate were invited again 2 years later, as were all those who tested negative and were still in the eligible age range. After 2018, gFOBt was replaced by a faecal immunochemical test (FIT), which has been shown to have marginally greater sensitivity and higher uptake rates than for gFOBt.^1^

Most of the published evidence for the efficacy of bowel cancer screening using gFOBt stems from a limited number of randomised controlled trials (RCTs).^2^, ^3^, ^4^, ^5^, ^6^ The largest of these trials, which commenced in the 1980s, was of 152,850 people aged 45–74 in the UK who were randomly allocated to gFOBt screening or no screening.^2^ This trial showed a 15% reduction in colorectal cancer (CRC) mortality among those who were invited for screening during an average follow‐up of 7.8 yrs.^2^ Smaller trials conducted in other countries have also demonstrated a reduction in CRC mortality from gFOBt screening.^3^, ^4^, ^5^, ^6^

RCTs remain the gold standard for assessing the benefit of screening interventions because the often marked differences between those who do and do not accept screening may not always be adequately controlled for in a non‐randomised setting. However, they are often limited in their power to assess differences in screening benefit by individual characteristics, or by cancer subtypes. Furthermore, RCTs measure the “efficacy” of screening, defined as the benefits of screening in an ‘ideal’ setting (e.g. in highly specialised centres), which may be greater than the “effectiveness” of screening, which measures the benefits of screening under routine delivery.^7^

The Million Women Study (MWS) is a large UK cohort of 1.3 million women who were recruited in 1996–2001 at the same time as their invitation for routine breast screening. Usually, observational studies of the effectiveness of cancer screening, based on those who did and did not attend screening, are severely limited by the substantial potential for residual confounding. However, linkage of the MWS to national bowel cancer screening data in the period 2006–2013, during which the screening programme was gradually rolled out, offers a unique opportunity for relatively unbiased assessment of the effectiveness of CRC screening in UK women by comparing CRC mortality in women who were and were not invited for bowel screening during that period.

We present here a comparison of characteristics in women who did and did not accept bowel screening among MWS participants born 1940–1950, who were all invited for screening (study A), and an assessment of the relationship between invitation to bowel screening and CRC mortality among MWS participants born 1935–1939 based on a quasi‐randomised trial design (study B).

## 2 METHODS

### 2.1 The Million Women Study

Between 1996 and 2001 around 1.3 million women (aged on average 56 yrs) joined the MWS cohort through NHS breast screening clinics in England and Scotland, completing a questionnaire about anthropometric, social, and demographic factors and other personal characteristics. The cohort has been resurveyed at approximately 3–5 yearly intervals since recruitment, and women were followed up for deaths, cancer registrations, and hospital admissions via electronic linkage to routinely collected NHS data.

### 2.2 Linkage to bowel screening data

**FIGURE 1:**
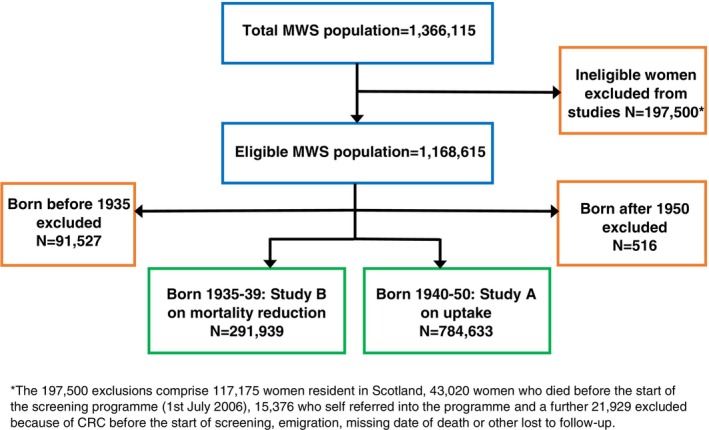
Flowchart showing MWS population with exclusions and the 1935–1939 and 1940–1950 years of birth subcohorts used in the studies.

**FIGURE 2:**
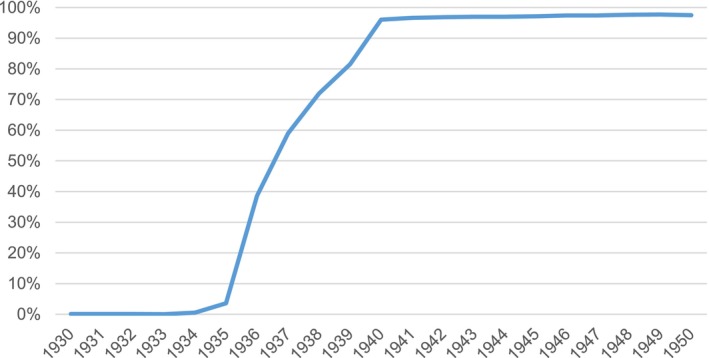
Proportion of MWS participants born 1930–1950 with a record of invitation to routine bowel screening, by year of birth.

In 2014, the MWS cohort was linked to the national bowel cancer screening database to obtain information on invitation and acceptance of bowel cancer screening among participants during the period up to the end of March 2013. After excluding women who had either died, had a CRC registration, or were lost to follow‐up before 1st July 2006 (the start of the screening programme), there were 1,168,615 women eligible for inclusion in our studies (Figure 1), of whom 78.6% had a record on the NHS BCSP database before the end of March 2013. The percentage of eligible women with at least one screening invitation before this date, by year of birth, is shown in Figure 2. Almost all women born before 1935 were not invited for screening. By contrast, virtually all women born from around 1940 onwards were invited for screening, with only 2% not receiving an invite because they had permanently opted out of the programme prior to receiving a screening kit, usually due to ill health. Women born in the years 1935–1939 are the sub‐cohort most affected by the gradual roll‐out, with women born in the later years being more likely to be invited (Figure 2). During the roll out, women were either invited or never invited based on geographical location and age. Some of the women born in this period received an invitation at age 68 or 69 yrs. and then received further invitations after the programme extended the age range up to 74 yrs. However, other women of a similar age at the start of the programme, but who resided in areas where the programme started later, did not receive any invitation to screening at all. The percentage of women who received an invitation during the period covered according to age at first invitation is shown in Figure A1 in supporting information.

The range of screening invitation experiences among women within the MWS allows an assessment of characteristics associated with screening acceptance in women born from 1940 onwards who were all invited, and an opportunity to assess the effect of screening invitation on CRC mortality in women born 1935–1939 who were or were not invited on the basis of age and region of residence.

### 2.3 Characteristics of women who did and did not accept an invitation for screening (study A)

Among women born 1940–1950 who received at least one screening invitation, we compared characteristics of those who accepted screening invitations (during the period 2006–2013) with those that did not, based primarily on acceptance of the first invitation to screening. We also examined characteristics in women who declined all invitations and those who accepted at least one invitation in that period. The characteristics studied were social deprivation (as measured by the Townsend deprivation index^8^), height, body mass index (BMI), smoking history, alcohol consumption, strenuous exercise, oral contraceptive use, hormone replacement therapy (HRT) use and family history of bowel cancer. We also compared CRC and all‐cause mortality between a woman's first invitation for screening up to the end of December 2021, in those that declined or accepted invitations. CRC deaths were defined as all deaths with any ICD code in the range C18–C20 as the underlying cause.

### 2.4 Analysis of mortality in women who were and were not invited for screening (study B)

While linkage of the MWS cohort to NHS BCSP data allows identification of all women who were invited for screening between 2006 and 2013, this information could not be used as a basis for assessing the impact of invitation for screening on mortality because the ~2% of women who permanently opted out of the programme before receiving a screening kit do not appear on the screening system database. If we were to define women as having been invited or not, purely on the basis of having a record on the screening database, such women would all be included in the uninvited group, which would seriously bias comparisons of mortality by screening invitation since they typically have a much higher death rate, particularly in the first few years of follow‐up.

In order to construct a quasi‐randomised trial design, therefore, the 291,939 MWS participants born 1935–1939, and potentially eligible for bowel cancer screening, were assigned to a “no intention to invite” and an “intention to invite” group based on area of residence and year of birth. Women in each of 72,650 individual combinations of 6‐month date of birth category and postcode sector were assigned to the “intention to invite group” if the great majority (>85%) of women in their date of birth/postcode category were invited to screening (according to the screening database), or to the “no intention to invite group” if the great majority of women in their date of birth/postcode category were not invited to screening. If between 15% and 85% of women in any given date of birth/postcode category were invited, women in this category were not included in the analysis. An example of how this allocation was done is given in Table A1 in supporting information. This method avoids a potentially serious bias due to differential misclassification of those women who opted out of screening as uninvited, but inevitably leads to a small degree of non‐differential misclassification in invitation status among the remaining women.

All women in both the “intention to invite” and “no intention to invite” groups were assigned a date of first screening invitation (or date of first pseudo screening invitation), on the basis of their actual date of screening invitation, for those who were invited, or the median date of screening invitation among women with the same 6‐month date of birth category and broad postcode category for those that were not. In order to assess the relative risk of all‐cause, and CRC‐related, mortality according to invitation group, women contributed person‐years of follow up from their date of first screening invitation (or date of first pseudo screening invitation) until loss to follow‐up, death, or end of follow‐up (31st December 2021).

Women who had died, were diagnosed with CRC, or lost to follow‐up before their date of first screening invitation (or date of first pseudo screening invitation) were excluded from analyses. Cox proportional hazards models with age as the underlying time variable were used to estimate the relative risk for all cause and CRC‐specific mortality in the “intention to invite” versus the “no intention to invite” group. Analyses were stratified by year of birth and adjusted for deprivation (tertiles), height (<160 cm, 160–164.9 cm, 165+cm), BMI (<25, 25–29, 30 kg/m^2^+), smoking (never, past, current), alcohol intake (0, 0.1–2 units per week [pw], 2.1–6.9 units pw, 7+ units pw), strenuous exercise (rarely/never, up to once/pw, once+ pw), oral contraceptive use (ever, never) and use of HRT (ever, never). The relative risk of CRC mortality was also examined by anatomical sub‐site and grade of the cancer. Location was defined from ICD‐10 codes as either proximal (right‐sided) colon [caecum (C180) to transverse colon (C184)], distal (left‐sided) colon [splenic flexure (C185) to sigmoid colon (C187)] or rectal [rectosigmoid junction (C19) to rectum (C20)]. Where CRC location at death was unspecified, CRC location was based on cancer registration data.

Based on data using FIT,^9^ the sojourn time (time from presymptomatic screen detectable to symptomatic disease) has been estimated at around 3–4 years for cancer, and 5–7 years for advanced adenoma, and these intervals are likely to be broadly similar for gFOBt. In the UK gFOBt randomised trial, differences in CRC mortality between the intervention and control groups were first evident from cumulative mortality graphs after around 3–4 years.^2^ We have therefore chosen to present our main findings based on comparisons of risk in the period 4 or more years from first screening invitation, although results are also presented for the entire follow‐up period and, separately, within the periods <4 years, 4–7 years, and 8+ years from first invitation date.

## 3 RESULTS

### 3.1 Characteristics of women who did and did not accept an invitation for screening (study A)

All women born from 1940 onwards, with the exception of those that had opted out of the programme, were invited for screening (Figure 2). Of the 784,633 women in our dataset born between 1940 and 1950, 1111 were excluded because they had died, had CRC diagnosed, or were lost to follow up before being invited. A further 22,891 were not on the NHS BCSP database and were assumed to have opted out of the programme prior to being registered. A further 8624 who appeared on the database, but were never sent a kit, were also considered to have opted out. Women who opt out of the programme often do so due to ill health, and so findings for these women are presented separately.

The characteristics of the remaining 752,007 women who were invited are shown in Table 1 according to whether or not they accepted their first invitation between 1st July 2006 and 31st March 2013. The table also includes details of the 31,515 women who opted out of screening. The 213,178 women who declined screening were likely to have higher BMI (26.8 kg/m^2^ vs. 25.9 kg/m^2^), to be current smokers (32.5% vs. 17.3%), and to be from more deprived areas (41.8% vs. 29.6% in most deprived tertile) than the 538,829 women who accepted screening. Nearly 80% of never smokers from the lowest tertile of deprivation accepted their first invitation for screening as compared with only around 50% of current smokers from the highest tertile of deprivation (Table 2).

**TABLE 1:** Characteristics of Million Women Study participants born 1940–1950 who did and did not accept an invitation for routine bowel screening.^a^

	Opted out of programme^a^	Declined 1st invite	Accepted 1st invite
Number of women	31,515	213,178	538,829
Mean age at 1st invitation (SD)	n/a	64.1 (2.8)	64.2 (2.8)
SES, % in most deprived tertile	34.2%	41.8%	29.6%
Height (cm), mean (SD)	162.3%	161.8 (6.8)	162.3 (6.6)
BMI (kg/m^2^), mean (SD)	26.2%	26.8 (5.2)	25.9 (4.5)
Smoking status, % current	29.0%	32.5%	17.3%
Alcohol intake, % 7+ units/week	26.9%	24.0%	26.1%
Strenuous exercise, % 1+/week	20.8%	19.3%	22.6%
Oral contraceptive use, % ever	67.3%	66.5%	69.4%
Menopausal hormone therapy use, % ever	55.6%	51.1%	55.6%
Family history of CRC (mother or father), %	10.0%	7.7%	8.9%
Mortality by end of 2021			
All deaths, number (%)	15,078 (47.8%)	44,647 (20.9%)	57,218 (10.6%)
CRC related deaths, number (%)	571 (1.81%)	1270 (0.60%)	1950 (0.36%)
Estimated number of screening invitations by end of 2021, mean (SD)	n/a	5.2 (1.6)	5.4 (1.4)

^a^ Comprises 22,891 women who had opted out of screening and were not on the database (of whom 56% had died by the end of follow‐up), and a further 8624 women who had opted out but were on the screening database (of whom 27% had died by the end of follow‐up).

*Note*: Uptake for 1st invite = 71.6% (538,829/752,007). Uptake from any invite between 1st July 2006 and March 31st 2013 = 79.2%. Uptake for 1st invite including all women who opted out of the screening programme = 68.8% (538,829/783,522).

**TABLE 2:** Proportion (%) of Million Women Study participants born 1940–1950 who accepted their first bowel screening invitation, by deprivation status and smoking status.

	Smoking history		
Deprivation tertile	Current	Past	Never
Most deprived	52.6 (38,231/72,676)	69.9 (44,793/64,070)	69.6 (66,496/95,557)
Middle	60.8 (27,327/44,937)	76.3 (51,401/67,336)	77.0 (92,892/120,588)
Least deprived	63.8 (22,386/35,118)	79.0 (54,098/68,495)	79.7 (109,275/137,078)

Women who declined their first bowel cancer screening invitation were almost twice as likely to subsequently die from any cause by the end of follow up (20.9% vs. 10.6%) (*p*‐value for heterogeneity < .001) and were 65% more likely to die specifically from CRC (0.60% vs. 0.36%) (*p*‐value for heterogeneity < .001).

The vast majority (92%) of women who accepted their first invitation also accepted their second invitation, and over two‐thirds (71%) of those who declined their first screening invitation also declined their second invitation. Thus patterns of characteristics in women who never accepted any invitation compared with those who accepted all invitations were broadly similar to those according to acceptance of the first invitation (data not shown). Among women who opted out of the screening programme before or after being registered on the NHS BSP, 56% and 27%, respectively, had died by the end of the follow‐up period, confirming that many of these women were probably in comparatively poor health at the time of invitation.

### 3.2 Analysis of mortality in women who were and were not invited for screening (study B)

The MWS women born 1935–1939 were allocated to the “intention to invite” or “no intention to invite” groups based on postcode sector and 6 month date of birth interval. Out of the 50,509 combinations of postcode sector and 6‐month date of birth interval which contained MWS participants, there were 22,403 in which the great majority of eligible women were invited for screening and 22,445 in which the great majority of women were not invited. There were a further 5661 where the proportion of women invited varied between 15% and 85%. The 44,848 combinations of postcode sector and date of birth interval in which women were either largely invited or not invited included a total of 254,016 women.

After exclusion of 7856 women who had died or had a CRC diagnosed before the date of first invitation (or date of first pseudo invitation), the final study groups comprised 111,956 women in the “no intention to invite” group and 134,204 women in the “intention to invite” group. As expected, the vast majority of women in the “intention to invite” group (98%) could be identified as having had a screening episode on the NHS BCSP database, and the vast majority of those in the “no intention to invite” group (99%) could not. MWS participants are broadly representative of UK women who attend for routine breast screening and the acceptance rate for gFOBt based bowel screening in this group was notably higher (70%) than that seen in the general population of women in England at this time (55%). The uptake for the “no intention to invite” group was also around 70%, although the number of women invited in this group was very small.

Table 3 shows the characteristics of women in the two invitation groups. Most women in the intention to invite group had 1–3 invitations before 31st March 2013 but a small number (7%) will have had one further invitation after that date which was included in the estimated number of invitations for that group. The mean number of invitations was 0.01 in the “no intention to invite” group, and 2.40 in the “intention to invite” group. The characteristics of women allocated to each group were similar except for a marginally higher level of deprivation and current smoking in the women in the intention to invite group. Of the women who tested gFOBt positive, 90% had diagnostic tests within the NHS system. Of these, 92% were colonoscopy, 3% flexi‐sig, 3% CT colonoscopy and 1% barium enema. Of the other 10%, some or all may have had private diagnostic tests but that data is not available.

**TABLE 3:** Characteristics of women by invitation group.^a^

	No intention to invite	Intention to invite
	*n* = 111,956	*n* = 134,204
Mean invitations	0.01	2.40
Age at 1st (pseudo‐)invite, mean, (SD)	73.2 (1.6)	70.7 (2.2)
Year of birth, mean (SD)	1936.2 (1.2)	1937.9 (1.0)
Year of 1st (pseudo) invite, mean	2009.4	2008.6
SES % in most deprived tertile	30.5%	36.4%
Height, cm mean, (SD)	162.0 (6.5)	161.9 (6.6)
BMI, kg/m^2^, mean, (SD)	26.4 (4.5)	26.4 (4.6)
Smoking, % current	13.6%	16.2%
Alcohol, % 15+ units/wk	3.77%	3.98%
Strenuous exercise, % 1+/wk	19.8%	19.2%
Oral contraceptive use, % ever	40.3%	45.0%
Menopausal hormone therapy use, % ever	41.6%	47.8%
Family history of CRC (mother or father)	8.5%	8.8%

^a^ Includes all women born 1935–1939 who had not died or been diagnosed with CRC prior to the date of their first invitation or pseudo date of first invitation.

During an average duration of follow‐up for mortality of 11.9 years, there were 73,646 deaths, of which 2048 were from CRC. Table 4 shows relative risks for all cause mortality, and CRC related mortality during the period four or more years after entry into the study. As might be expected, given that the groups were broadly comparable in all respects other than invitation for bowel screening and that analyses were adjusted for potential confounders, there was no evidence of any difference in all‐cause mortality between the intention to invite group and the no intention to invite group. Nor was there any significant difference in the risk of overall CRC mortality between the two groups (RR = 0.94, 95%CI 0.83–1.06). When CRC mortality was examined by tumour sub‐site, there was a significant reduction in risk of death from distal colon cancers (RR = 0.64, 95%CI 0.47–0.88) but not from proximal colon cancers (RR = 1.02, 95%CI 0.83–1.25) or rectal cancers (RR = 0.97, 95%CI 0.79–1.20) (*p*‐value for heterogeneity by sub‐site = .05). There was no significant difference in the relative risk of CRC mortality according to tumour grade (*p*‐value for heterogeneity by grade = .44).

**TABLE 4:** Relative risk of all cause and CRC‐related mortality in the period 4 or more years after entry into the study, among women in the intention to invite group compared with those in the no intention to invite group.

	*N* = 231,140	Unadjusted RR (95%CI)	Adjusted RR (95%CI)
Cause of death			
All deaths			
Not invited	28,641	Reference	Reference
Invited	30,167	1.03 (1.01,1.05)	1.00 (0.98,1.02)
All CRC deaths			
Not invited	791	Reference	Reference
Invited	858	1.00 (0.90,1.11)	0.94 (0.83,1.06)
Proximal colon			
Not invited	286	Reference	Reference
Invited	318	1.03 (0.87,1.22)	1.02 (0.83,1.25)
Distal colon			
Not invited	132	Reference	Reference
Invited	125	0.71 (0.55,0.93)	0.64 (0.47,0.88)
Rectum			
Not invited	270	Reference	Reference
Invited	309	1.06 (0.89,1.26)	0.97 (0.79,1.20)
CRC: Grade 1&2			
Not invited	267	Reference	Reference
Invited	293	0.84 (0.70,1.00)	0.85 (0.69,1.05)
CRC: Grade 3&4			
Not invited	100	Reference	Reference
Invited	119	0.82 (0.62,1.09)	1.02 (0.72,1.44)

*Note*: NB CRC location unknown for similar proportions in both groups—103 (12.4%) of not invited and 106 (13.0%) of invited.

Analyses of overall and CRC mortality risk during the entire follow‐up period, and during the periods 0–3, 4–7, and 8+ years after first screening are shown in Table A2 in supporting information. As might be expected, no differences in CRC mortality risks were evident in the first 3 years after first screening. Nor were there any clear differences in CRC mortality risk in the period 4–7 years after screening. In the interval 8 or more years after first screening, there was still no difference in overall CRC mortality, but there was evidence of a difference in CRC mortality risk by sub‐site with a material reduction in risk of distal colon cancers (0.52, 0.34–0.78) but not proximal colon cancers (0.95, 0.73–1.23) or rectal cancers (0.93, 0.70–1.22) (*p*‐value for heterogeneity = .03).

## 4 DISCUSSION

This large observational study analysed data from nearly a million (998,167) women in England born 1935–1950, who received differing numbers of invitations for routine bowel screening. We found that among women born 1940–1950 (study A), all of whom were invited for screening, those who declined screening were more likely to be from deprived areas, to be heavy smokers, and had a substantially greater risk of CRC mortality. Among women born 1935–1939 (study B), only some of whom were invited for screening, we did not observe any clear reduction in the risk of CRC mortality in the period 4 or more years from first invitation in women who received an average of 2.40 screening invitations compared with those who received 0.01 invitations at ages 68–74 years. Nor did we observe any significant reduction in the risk of CRC death due to proximal colon or rectal cancers associated with invitation to screening, although we did observe a moderate (36%) reduction in the risk of mortality from distal colon cancers.

Studies of screening uptake within the NHS BCSP have shown that there are notable disparities in socioeconomic status between those who do and do not attend for bowel screening.^10^ Our study also demonstrated substantial and significant differences in the prevalence of smoking and other risk factors for CRC. While non‐acceptance of bowel cancer screening may have contributed somewhat to the 65% greater risk of CRC mortality in those who declined screening, the magnitude of this excess risk indicates that those who decline screening are precisely the group who, in theory, should benefit most from screening. While replacement of gFOBt with FIT has been shown to increase uptake of screening by around 6%,^1^ there are likely to be broadly similar differences in women who do and do not accept invitations for FIT screening.

A meta‐analysis of the randomised evidence regarding the efficacy of biennial gFOBt bowel cancer screening (with endoscopic follow up of positive test results)^11^ found a reduction in overall mortality from CRC of 12% (95%CI 6%–17%), with no evidence of any difference in effect for women (17%, 3%–29%) and men (16%, 2%–27%). Our findings, based on some 2048 CRC deaths, failed to show a significant reduction in risk of CRC mortality among women in the period 4 or more years after first screening invite (6%, −6% to 17%), although the non‐significant reduction in risk observed in invited women (11%, −4% to 25%) in the period 8 or more years after the first screening invite is consistent with the estimate for women from the meta‐analysis in which all studies had a median follow up of at least 14 years.

Unlike the meta‐analysis, which did not detect any difference by anatomical sub‐site (proximal colon, distal colon, or rectum), our findings suggest that any reduction in CRC mortality in these women may be due largely to a reduction in mortality from distal colon cancers. There are several possible reasons for the apparent discrepancy between our findings and those of the meta‐analysis. Firstly, the difference in the average number of screening invitations between the comparison groups considered here (2.4) is similar to that in one randomised trial^4^ but lower than that in others,^3^, ^5^ although the findings from the randomised data did not appear to differ by number of screening rounds. The discrepancy is unlikely to be due specifically to differences in screening uptake between our study and the trials since screening uptake in this group of women was ~70%, which is higher than the average attendance in most trials. It is perhaps more likely that the lesser effect of screening invitation on overall CRC mortality seen here is due to the fact that our cohort was exclusively women. While the pooled analysis of trial data found no evidence of a difference in CRC mortality benefit for men and women, an updated analysis of one of the trials,^12^ and two population‐based studies^13^, ^14^ have reported lower reductions in CRC mortality among invited women than men, although one further observational study did not.^15^ The latter population‐based studies were based on greater numbers than the randomised trials, and have considerably more power to detect differences in effects by sex and sub‐site. Of these studies only the Finnish study^13^ was comparable in design to our study. It has been documented that women are more likely than men to develop proximal than distal cancers,^16^ and we and others^17^, ^18^ have previously shown that gFOBt sensitivity is lower for proximal than distal cancers. Taken together, these differences could have contributed to the lack of an overall reduction in risk of CRC mortality observed here. Our findings may also partly reflect the fact that any impact of screening within a national population‐wide programme is likely to be less than that observed in the context of a randomised clinical trial (with possibly greater clinical expertise).

There is biological plausibility for differences in the effect of screening by sub‐site. In particular, rectal cancers may be less likely to be detected at gFOBt screening because haemoglobin from rectal cancers is more likely to remain within intact erythrocytes and therefore not detectable by un‐rehydrated gFOBt. In addition, haemoglobin from cancers in the proximal colon has more time to degrade than that from the distal colon.^18^ If there are such differences in the sensitivity of gFOBt by sub‐site, this could have contributed to the lesser effect of screening on CRC mortality observed here because of the relatively higher incidence of proximal colon than distal colon cancers in women.^15^ Screening sensitivity may also be reduced in women because of reduced compliance with the next stage of screening in those with a positive gFOBt test, as it has been reported that women are less likely to tolerate colonoscopy procedures.^19^ It is also possible that people with rectal cancer may present symptomatically at an earlier stage than those with colon cancer and may therefore not benefit so much from screening.

### 4.1 Strengths and limitations of our study

The main limitation of this study is that it is not a randomised trial and instead, allocation of women to the “intention to invite” or “no intention to invite” group was based on the likelihood that they were invited for screening during the roll out period, given their postcode and date of birth. While this method does inevitably lead to some small degree of misclassification of women's invitation status, virtually all of those in the intention to invite group were identified on the NHS BCSP database as having at least one screening invitation, and virtually all of those in the no intention to invite were not, suggesting that any degree of misclassification was small. The study design used here (i.e. a quasi‐randomised trial) should result in comparison groups that are similar in all respects other than invitation status and this was broadly true, apart from some slight differences in age and deprivation status which were probably due to earlier roll‐out of the programme to specific ages and regions. While these small differences would be unlikely to materially affect the findings we adjusted our analysis for year of birth, deprivation and other known risk factors for CRC^20^ to further minimise the potential for confounding. We were also only able to assess the impact of an average of around 2.4 invitations between ages 68–74 years, but screening uptake in this cohort was high (~70%) and so the effect on CRC mortality of two screening invitations in this cohort might be expected to be equivalent to the effect of slightly more screening invitations in studies where uptake is around 55%–65% (as was typical in previous randomised trials). Another limitation of the study is that it was only able to assess the impact of gFOBt screening on CRC mortality, and did not provide information on the effect of FIT screening which replaced gFOBt screening in 2018. The main advantages of this study are that it was able to assess the effectiveness of routine screening as delivered at a population level as opposed to within a clinical trial, and that it provides a much larger sample size to address questions about the impact of screening on CRC mortality in women, and by cancer subtype.

### 4.2 Implications for bowel cancer screening with FIT

Our findings are particularly important given that the programme is currently being extended to include screening of men and women aged 50–59 on the basis of modelling evidence which did not take account of potential differences in CRC mortality reductions between men and women.^21^ The findings of the FIT pilot study indicated an increased uptake from 62.1% to 68.1% for women and an estimated increased positivity from 1.5% to 1.6% at a cut‐off of 120 ug/g faeces.^1^ These are modest gains, and recent studies of interval cancers arising from FIT screening,^22^, ^23^ showing higher risks of interval cancers in women, and in those with distal and rectal locations, suggest that FIT may have similar limitations to gFOBt. Further studies of interval cancers and mortality following FIT screening are, therefore, required, as well as studies aimed at assessing the utility of using sex‐specific cut offs for FIT screening.

### 4.3 Conclusions

Our findings show that UK women who accepted routine gFOBt screening between 2006 and 2013 were much less likely to have many of the known risk factors for CRC than those who declined screening. Our findings also show that, among women, routine bowel screening using gFOBt is associated with a clear reduction in deaths from distal colon cancer, but not necessarily from proximal colon or rectal cancer. While overall acceptance and screening sensitivity for FIT have been shown to be greater than for gFOBt, further research into the effectiveness of FIT‐based screening by sex, tumour subtype, and location is warranted.

## AUTHOR CONTRIBUTIONS

**Roger G. Blanks:** Conceptualization; methodology; formal analysis; writing – original draft; writing – review and editing. **Rupert J. Alison:** Conceptualization; data curation; methodology; formal analysis; writing – review and editing. **Gillian K. Reeves:** Conceptualization; methodology; writing – review and editing.

## FUNDING INFORMATION

This work was supported by Cancer Research UK [grant number C16077/A29186].

## CONFLICT OF INTEREST STATEMENT

The authors declare no conflicts of interest.

## ETHICS STATEMENT

All study participants gave written consent to take part in the study, and ethical approval was provided by the Oxford and Anglia (now East of England‐Cambridge South) Multi‐Centre Research Ethics Committee (Ref: REC 97/5/01).

## Supporting information


**Appendix S1:** Supporting information.

## Data Availability

Anonymised data used here can be accessed by application to the investigators. The Million Women Study Data Access Policy can be viewed at [millionwomenstudy.org/data_access]. Further information is available from the corresponding author upon request.
