# Supplementary material for: Invitation for FOBt screening and colorectal cancer mortality: A prospective analysis in the Million Women Study cohort
Source: Int J Cancer. 2025 Apr 11;157(5):867–75. doi: 10.1002/ijc.35437 (PMC12232529; doi:10.1002/ijc.35437)

Invitation for FOBt screening and colorectal cancer mortality: a prospective analysis in the Million Women Study cohort

Roger G Blanks, Rupert J Alison and Gillian K Reeves

Table of Contents

Supplementary Table A1

Supplementary Table A2

Supplementary Figure A1

Table A1 – Gridpoints using half year of birth for postcode sector ‘BS4 1’

Half year of birth	% with an invitation date	Intention to invite
1935.0	0.0%	**No**
1935.5	0.0%	**No**
1936.0	0.0%	**No**
1936.5	0.0%	**No**
1937.0	0.0%	**No**
1937.5	0.0%	**No**
1938.0	0.0%	**No**
1938.5	11.1%	**No**
1939.0	100%	**Yes**
1939.5	100%	**Yes**
**TOTAL**	**26.3%**	

Table A1 shows the distribution of MWS participants who were born 1935-39 in the ‘BS4 1’ postcode sector according to 6-month year of birth interval, invitation status on the NHS BSP database, and allocated invitation group. If >85 of women in a given 6-month year of birth interval were or were not invited for screening (according to the NHS BSP database) then we allocated all women in that year of birth interval to the “intention to invite” or the “no intention to invite“ group, respectively. The sensitivity of the method to use of postcode district or postcode sector was tested and the postcode sector method was found to give better discrimination.

Table A2 - Relative risk of all cause and CRC-related mortality among women in the intention to invite group compared with those in the no intention to invite group by follow up period

	Follow up period from date of first invitation							
	<4 years		4-7 years		8+ years		Entire follow-up	
	N= *246,160*	**Adjusted RR (95% CI)**	***N=*** *231,140*	**Adjusted RR (95% CI)**	***N=*** *208,970*	**Adjusted RR (95% CI)**	***N=*** *246,160*	**Adjusted RR (95% CI)**
**Deaths**								
**All deaths**<br>Not invited<br>Invited	 *7,594*<br>*7,237*	 *reference*<br>0.98<br>(0.94,1.02)	 *11,459*<br>*10,549*	 *reference*<br>0.98<br>(0.94,1.01)	 *17,182*<br>*19,618*	 *reference*<br>1.01<br>(0.98,1.04)	 *36,238*<br>*37,408*	 *reference*<br>0.99<br>(0.97,1.01)
**All CRC deaths**<br>Not invited<br>Invited	 *202*<br>*197*	 *reference*<br>0.95<br>(0.74,1.22)	 *312*<br>*314*	 *reference*<br>0.99<br>(0.81,1.22)	 *479*<br>*544*	 *reference*<br>0.89<br>(0.75,1.04)	 *993*<br>*1,055*	 *reference*<br>0.95<br>(0.85,1.07)
**Proximal colon**<br>Not invited<br>Invited	 *67*<br>*66*	 *reference*<br>0.95<br>(0.60,1.49)	 *106*<br>*114*	 *reference*<br>1.03<br>(0.73,1.45)	 *180*<br>*204*	 *reference*<br>0.95<br>(0.73,1.23)	 *353*<br>*384*	 *reference*<br>1.03<br>(0.85,1.24)
**Distal colon**<br>Not invited<br>Invited	 *33*<br>*32*	 *reference*<br>1.04<br>(0.57,1.91)	 *53*<br>*56*	 *reference*<br>0.81<br>(0.48,1.35)	 *79*<br>*69*	 *reference*<br>0.52<br>(0.34,0.78)	 *165*<br>*157*	 *reference*<br>0.75<br>(0.57,0.99)
**Rectum**<br>Not invited<br>Invited	 *72*<br>*68*	 *reference*<br>0.93<br>(0.61,1.42)	 *117*<br>*112*	 *reference*<br>1.01<br>(0.72,1.41)	 *153*<br>*197*	 *reference*<br>0.93<br>(0.70,1.22)	 *342*<br>*377*	 *reference*<br>0.96<br>(0.80,1.16)
**CRC Grade 1,2**<br>Not invited<br>Invited	 *85*<br>*76*	 *reference*<br>0.95<br>(0.64,1.42)	 *128*<br>*132*	 *reference*<br>1.10<br>(0.80,1.51)	 *139*<br>*161*	 *reference*<br>0.66<br>(0.49,0.88)	 *352*<br>*369*	 *reference*<br>0.89<br>(0.74,1.07)
**CRC Grade 3,4**<br>Not invited<br>Invited	 *31*<br>*41*	 *reference*<br>1.20<br>(0.66,2.16)	 *51*<br>*60*	 *reference*<br>0.88<br>(0.54,1.44)	 *49*<br>*59*	 *reference*<br>1.08<br>(0.64,1.82)	 *131*<br>*160*	 *reference*<br>1.05<br>(0.78,1.42)

Fig A1 - Proportion of Million Women Study participants with a first invitation to screening at age 60-69, or at age 70-74, by calendar year

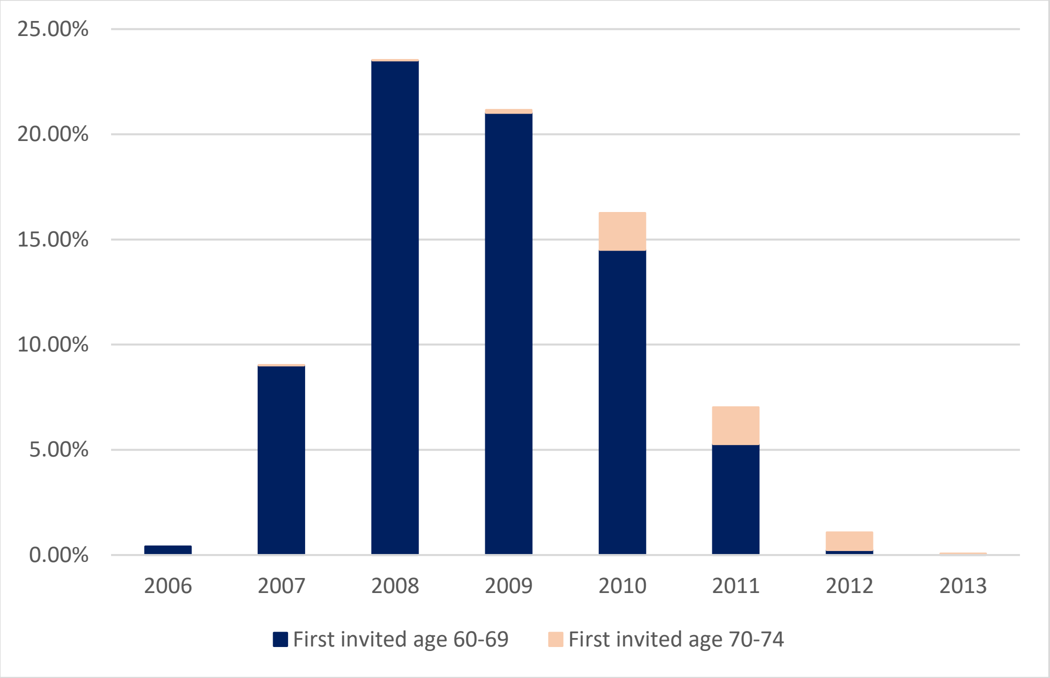

Supplement: Supplementary file 1 — Appendix S1: Supporting information. [file IJC-157-867-s001.pdf]
